# Ambient carbon monoxide and cardiovascular-related hospital admissions: A time-series analysis

**DOI:** 10.3389/fphys.2023.1126977

**Published:** 2023-03-08

**Authors:** Marzieh Taheri, Fatemeh Nouri, Mahdi Ziaddini, Katayoun Rabiei, Ali Pourmoghaddas, Sheikh Mohammed Shariful Islam, Nizal Sarrafzadegan

**Affiliations:** ^1^ Isfahan Cardiovascular Research Center, Cardiovascular Research Institute, Isfahan University of Medical Sciences, Isfahan, Iran; ^2^ Heart Failure Research Center, Cardiovascular Research Institute, Isfahan University of Medical Sciences, Isfahan, Iran; ^3^ Student Research Committee, Department of Occupational Health, Isfahan University of Medical Sciences, Isfahan, Iran; ^4^ Hypertension Research Center, Cardiovascular Research Institute, Isfahan University of Medical Sciences, Isfahan, Iran; ^5^ Interventional Cardiology Research Center, Cardiovascular Research Institute, Iran Isfahan University of Medical Sciences, Isfahan, Iran; ^6^ Institute for Physical Activity and Nutrition, Deakin University, Melbourne, VI, Australia

**Keywords:** carbon monoxide, hospital admissions, cardiovascular disease, ischemic heart disease, heart failure, cerebrovascular disease

## Abstract

**Background and aims:** Although several studies have investigated the association between air pollutants and cardiovascular diseases (CVDs) in recent years, a lack of evidence exists regarding carbon monoxide (CO) exposure, especially in the Eastern Mediterranean’s polluted regions. In this study, we aimed to evaluate the short-term effect of CO exposure on daily CVD hospital admissions in Isfahan, a major city in Iran.

**Methods:** Data were extracted from the CAPACITY study on daily CVD hospital admissions in Isfahan from March 2010 to March 2012. The 24-h mean CO concentrations were obtained from four local monitoring stations. In a time-series framework, the association between CO and daily hospitalizations for total and cause-specific CVDs in adults (ischemic heart disease (IHD), heart failure (HF), and cerebrovascular disease) was conducted using Poisson’s (or negative binomial) regression, after adjusting for holidays, temperature, dew point, and wind speed, considering different lags and mean lags of CO. The robustness of the results was examined *via* two- and multiple-pollutant models. Stratified analysis was also conducted for age groups (18–64 and ≥65 years), sex, and seasons (cold and warm).

**Results:** The current study incorporated a total of 24,335 hospitalized patients, (51.6%) male with a mean age of 61.9 ± 16.4 years. The mean CO concentration was 4.5 ± 2.3 mg/m³. For a 1 mg/m^3^ increase in CO, we found a significant association with the number of CVD hospitalizations. The largest adjusted percent change in HF cases was seen in lag0, 4.61% (2.23, 7.05), while that for total CVDs, IHD, and cerebrovascular diseases occurred in mean lag2–5, 2.31% (1.42, 3.22), 2.23% (1.04, 3.43), and 5.70% (3.59, 7.85), respectively. Results were found to be robust in two- and multiple-pollutant models. Although the associations changed for sex, age groups, and seasons, they remained significant for IHD and total CVD, except for the warm season, and for HF, except for the younger age group and cold seasons. Additionally, the exposure–response relationship curve of the CO concentrations with total and cause-specific CVD admissions showed non-linear relationships for IHD and total CVDs.

**Conclusions:** Our results showed that exposure to CO contributed to an increase in the number of CVD hospitalizations. The associations were not independent of age groups, season, and sex.

## 1 Introduction

The Global Burden of Disease (GBD) study demonstrated that cardiovascular diseases have been the leading cause of death since 1980 (Yang et al., 1998; Amini et al., 2021) and is one of the chronic, non-communicable diseases responsible for more than 12% of the global disease burden (Sadeghi et al., 2017). Most of the CVD deaths and global CVD burden occurred in low- and middle-income countries, particularly in the Eastern Mediterranean region (EMR) (Sarrafzadegan and Mohammmadifard, 2019; Baumgartner et al., 2020). Additionally, the rates of cardiovascular and cerebrovascular diseases are increasing in developing countries (Amini et al., 2021). According to GBD study 2015, Iran was one of the countries with the highest CVD rate in the world (Sarrafzadegan and Mohammmadifard, 2019).

An ever-increasing rate of urbanization, modernization, and industrialization in big cities in recent decades has increased the problem of air pollution (Chen et al., 2022). In recent years, numerous studies have investigated the human-induced hazardous effect of air pollution (Collart et al., 2018; Rodríguez et al., 2022). According to the World Health Organization (WHO), ambient air pollution is one of the most critical causes of death and disease, contributing to about 7.6% of total global deaths (Choi et al., 2019). Air pollution is the leading environmental risk factor for global health and the fourth largest risk factor for global mortality (Roth et al., 2020).

Carbon monoxide (CO) is one of the six air pollutants according to the National Ambient Air Quality Standards set by the American Environmental Protection Agency (Reboul et al., 2017). CO is a colorless, odorless, and tasteless gaseous pollutant produced by the incomplete combustion of fossil fuels like motor vehicles and has increased particularly in overcrowded traffic zones such as highways (Li et al., 2018). It has been observed that daily exposure to ambient CO is linked with prolonged hospital admissions, specifically for people with CVDs (Vahedian et al., 2017).

According to the results of the meta-analysis, a 1 ppm increase in CO concentration was associated with a 3.52% (0.52–4.54) increase in hospitalization or death related to heart failure (HF), which is an escalating public health issue that affects more than 23 million people worldwide (Shah et al., 2013). Another meta-analysis showed that for a 1 mg/m^3^ increase in CO concentration, the pooled risk ratio of myocardial infarction was 1.052 (95% CI 1.017–1.089) (Lee et al., 2020). Most of the air pollution-related deaths in 2016 occurred in low- and middle-income countries worldwide (Dastoorpoor et al., 2021). However, very few studies were conducted in these countries (Lee et al., 2020).

Like many other parts in Asia, the expansion of cities, rapid economic development, industrialization, and traffic congestion have led to intense air pollution in Iran. Isfahan is one of the most polluted cities in Iran for the following reasons: existence of multiple vehicles in the city, vast deserts in the nearby areas, and so many enormous industries in the suburb (Abdolahnejad et al.,2017). In Iran, 5.3%–10.7% of total deaths in four major cities (Ahvaz, Isfahan, Shiraz, and Tehran) were attributed to short-term exposure to air pollution (Mansouri and Hamidian, 2013; Jalali et al., 2021). In recent years, Isfahan, one of the largest cities in Iran, confronted numerous cases of CVDs. For almost all CVDs, a considerably high incidence rate was observed in subjects who participated in the Isfahan Cohort Study (ICS) (Talaei et al., 2013). Nouri et al. (2020) have shown a significant increasing long-term temporal trend in the incidence rate (IR) of IHD in the Isfahan population during a 15-year period. Although people in Isfahan encounter a high exposure to air pollution, specifically CO, and therefore a high risk of CVDs, a lack of studies investigating the effect of CO on CVDs in Isfahan is noticeable to the best of our knowledge. We aimed to study the relationships between total and cause-specific CVD daily hospital admissions and CO concentration in a short-term exposure period in Isfahan City, Iran. For this purpose, we used the data extracted from the CAPACITY (Correlation of Air Pollution with hospitalization And mortality of Cardiovascular and respIraTorY diseases) study, which is one of the few large-scale studies in the province designed to evaluate the effects of ambient air pollutants on cardiovascular and respiratory hospitalization and mortality.

## 2 Methods

### 2.1 Data

This study was conducted in Isfahan, the third metropolis located in the central part of Iran extending about 107,000 km^2^ with a population of more than 2.2 million. In a time-series framework, we investigated the association between ambient CO exposure and total daily hospital admissions for total and cause-specific CVDs in a short-term period.

Data were extracted from the CAPACITY study, which was approved by the Regional Bioethics Committee of Isfahan University of Medical Science and performed in Isfahan, Iran, from 21 March 2010 to 20 March 2012 (2 years in the Persian calendar).

In summary, the aim of the CAPACITY study was to evaluate the relationship between air pollutant indices and mortality or hospitalization regarding CVDs or pulmonary diseases. In this regard, the hospital admissions related to cardiovascular or respiratory diseases of patients who lived in Isfahan were registered in 15 out of 16 hospitals that were eligible to admit patients with CVDs or respiratory diseases. Mortality data for the same period were obtained from Isfahan’s cemetery. The data from the International Classification of Diseases Tenth Revision (ICD-10) including I00-I99 and J00-J99 were used for cardiovascular and respiratory problems, respectively.

The air pollution data including data on pollution due to SO_2_, NO_2_, CO, O_3_, PM_10_, and PM_2.5_ (for 10 months) were gathered from four traffic stations operated by Isfahan’s Department of Environment. The meteorological data on temperature, wind speed, and dew point were gathered from the Isfahan Weather Forecast Organization. More details on data collection and quality control procedures were described elsewhere (Rabiei et al., 2017; Karbakhsh et al., 2022).

In this study, ischemic heart diseases (I20–I25), heart failure (I50), and cerebrovascular diseases (I60–I69) were considered cause-specific CVDs. These diseases and any other CVDs including hypertensive heart disease (ICD10: I10–I15), conduction disorders and blocks (I44–I45), cardiac arrest (I46), arrhythmias (I47–I49), and other unspecified disorders of the circulatory system (I99) were evaluated as total CVDs. Although air pollution measurements were recorded on an hourly basis, the current study used an average of 24-h exposure across all stations.

### 2.2 Statistical analysis

The frequency and percentage of total and cause-specific CVDs according to age groups, sex, and season were reported. Additionally, mean ± standard deviation and the quartile of air pollution and meteorological data were reported.

To investigate the effect of CO exposure on CVDs, we used different types of generalized linear models wherever appropriate.

First, considering the suggestions of other studies such as Li et al. (2018) and Xu et al. (2021), single pollutant models were derived from 0 to 5 lag days, and multiple-day moving averages of lags including lag0–1, lag2–5, and lag0–5. For instance, lag0–1 stands for the average concentrations of CO in lag0 and lag1. Also, all models were repeated after adjusting for holidays, temperature, dew point, and wind speed. Then, the most effective lag period for each disease was identified, and further analyses were performed.

In addition, two-pollutant models were applied to validate the robustness of the CO results after adjustment for other pollutants such as PM_10_, NO_2_, SO_2_, and O_3_. Moreover, results were validated in multiple-pollutant models. To reduce the collinearity between CO and other pollutants, other pollutants were combined *via* principal component analysis (PCA).

Additionally, subgroup analysis was conducted using a multi-pollutant model within age groups (18–64 years and ≥65 years), sex (female and male), and season (22nd of March to 25th of September as the warm season and other days as the cold season) with adjustment for meteorological data and holidays. Furthermore, subgroups were compared using Fisher’s Z-statistics and normal distribution as shown in the following equation:

$$Z=\left(\beta_{1}-\beta_{2}\right)/\sqrt{{{SE}_{1}}^{2}+{{SE}_{2}}^{2}}.$$

Here, $\beta_{1}$ and $\beta_{2}$ represent the estimates of the two categories, and SE_1_ and SE_2_ represent their respective standard errors.

The types of generalized linear models (GLMs) that were performed depended on the existence of zero truncation and overdispersion problem. For more explanation, the GLM for each disease was as follows: for HF and cerebrovascular disease, Poisson’s regression approach was assessed in all models including subgroup analysis of age, sex, and season. For IHD, in total population and subgroup analysis of seasons and the elderly age group, as the number of days without any admission was 0 and overdispersion existed, zero-truncated negative binomial regression analysis was carried out, whereas in both male and female patients and the younger age group, negative binomial regression analysis was performed. For total CVDs, in total population and subgroup analysis, the zero-truncated negative binomial regression was carried out.

The linearity of relationships between CO concentrations and the relative risk (RR) of total and cause-specific CVDs was examined using cubic splines with 2–6 knots (Croxford, 2016). For each disease, the best number of knots was selected according to Akaike’s information criterion (AIC), and the likelihood ratio test (LRT) was performed to check the existence of non-linear effects.

Additionally, the exposure–response relationship curves of CO and total and cause-specific CVDs were drawn.

The relative risk (RR) was computed through Poisson’s regression or negative binomial regression model using the following formula: RR = exp(β), where β represents the regression coefficient related to CO exposure. We reported the percentage changes (PC% = [RR-1] × 100%) in daily hospital admissions for CVDs per 1 mg/m^3^ increase in CO concentrations with the corresponding 95% confidence interval (CI).

All the data analyses were conducted in Stata statistical software (version 14; Stata Corp., College Station, TX, United States). A *p*-value less than 0.05 was considered statistically significant.

## 3 Results

In this study, a total of 24,335 daily hospital admissions related to one of the CVDs were investigated for patients aged more than 18 years (mean age 61.9 ± 16.4 years). Of these, 12,960 had IHDs (53.3%), 2,698 had cerebrovascular diseases (11.1%), and 1,347 had HF (5.5%). The number of all CVD and IHD cases in daily hospital admissions was higher in the younger age group (<65 years) and slightly higher in the cold season. However, the number for HF and cerebrovascular disease cases was higher in the elderly age group (>=65 years) and slightly higher in the warm season. For total and cause-specific CVDs, admissions were slightly higher among men (Table 1).

**TABLE 1:** Number (percentage) of daily hospital admissions for total and cause-specific CVD stratified by age groups, sex, and seasons.

	Total CVD (24,335)	IHD (12,960 (53.3%))	HF (1,347 (5.5%))	Cerebrovascular disease (2,698 (11.1%))
Age				
18–64	12,969 (53.3)	7,933 (61.2)	459 (34.1)	869 (32.2)
≥65	11,366 (46.7)	5,027 (38.8)	888 (65.9)	1829 (67.8)
Sex				
Female	11,775 (48.4)	5,923 (45.7)	581 (43.1)	1,283 (47.6)
Male	12,560 (51.6)	7,037 (54.3)	766 (56.9)	1,415 (52.4)
Season				
Warm	11,839 (48.7)	6,484 (49.9)	690 (51.2)	1,370 (50.8)
Cold	12,496 (51.3)	6,476 (50.1)	657 (48.8)	1,328 (49.2)

CVD, Cardiovascular disease; IHD, ischemic heart disease; HF, heart failure.

Average daily concentrations of CO, SO_2_, NO_2_, O_3,_ and PM_10_ were 4.5 ± 2.3 mg/m^3^, 37.6 ± 28.5 ppb, 40.8 ± 20.6 ppb, 30.8 ± 12.9 ppb, and 139.1 ± 52.2 μg/m^3^, respectively. Also, the average of meteorological data, namely, wind speed, temperature, and dew point was 5.2 ± 2.4 km/h, 60.4 °F± 18.5°F, and 28.8°F ± 8.9°F, respectively (Table 2).

**TABLE 2:** Descriptive statistics for air pollution concentrations and weather conditions.

	Mean ± SD	Median (Q1–Q3)
CO	4.5 ± 2.3	4 (2.7–35.5)
SO_2_	37.6 ± 28.5	28.5 (21.4–40.7)
NO_2_	40.8 ± 20.6	35.4 (29.6–50.4)
O_3_	30.8 ± 12.9	30.9 (22.8–38.6)
PM_10_	139.1 ± 52.2	149.7 (97.0–170.2)
Wind speed	5.2 ± 2.4	4.9 (3.6–6.2)
Temperature (F)	60.4 ± 18.5	61.9 (42.1–77.4)
Dew point (F)	28.8 ± 8.9	29.7 (22.4–35.5)

CO, carbon monoxide; SO_2_, sulfur dioxide; NO_2_, nitrogen dioxide; O_3_, ozone; PM_10_, Particulate matter with an aerodynamic diameter <10 μm.

Table 3 shows the crude and adjusted effect of CO exposure (1 mg/m^3^ increase) with various lag patterns on the percentage change in daily hospital admission for IHD, HF, cerebrovascular disease, and total CVDs. We found that ambient CO was positively and significantly associated with the percentage change in daily hospital admissions for total and cause-specific CVDs except lag periods 2 and 3 which were not significant for HF in the adjusted models. For HF, in the adjusted models, the most effective lag period was lag0 with a percent change of 4.61 (2.23, 7.05), while for admissions with IHD, cerebrovascular disease, and total CVDs, the biggest increase was observed in the cumulative mean value of lag2–5 with a percent change of 2.23(1.04,3.443), 5.7(3.59,7.85), and 2.31 (1.42,3.22), respectively. Therefore, the following analyses only focused on the lag period 0 for HF and the moving average of lag2–5 for others.

**TABLE 3:** Crude and adjusted percent change in daily hospital admissions in total and cause-specific CVDs per 1 mg/m^3^ CO increase.

	Crude				Adjusted			
	IHD	HF	Cerebrovascular disease	Total CVD	IHD	HF	Cerebrovascular disease	Total CVD
Lag0	1.75 (0.72,2.79)	4.33 (1.99,6.72)	4.40 (2.74,6.08)	1.87 (1.04,2.71)	1.26 (0.27,2.26)	**4.61 (2.23,7.05)**	4.49 (2.76,6.17)	1.66 (0.90,2.42)
Lag1	1.48 (0.45,2.53)	3.30 (0.97,5.69)	3.75 (2.09,5.43)	1.61 (0.78,2.45)	1.23 (0.22,2.25)	2.89 (0.51,5.33)	3.89 (2.29,5.69)	1.43 (0.67,2.21)
Lag2	1.98 (0.95,3.02)	3.03 (0.69,5.42)	3.48 (1.82,5.16)	1.76 (0.93,2.59)	1.77 (0.77,2.78)	2.22 (-0.17,4.67)	3.45 (1.75,5.17)	1.45 (0.68,2.45)
Lag3	1.85 (0.81,2.89)	2.39 (0.06,4.79)	4.16 (2.51,5.85)	1.78 (0.94,2.62)	1.64 (0.64,2.66)	2.14 (-0.26,4.59)	4.27 (2.58,5.99)	1.63 (0.86,2.39)
Lag4	2.01 (0.97,3.05)	3.64 (1.31,6.04)	3.58 (1.92,5.26)	1.96 (1.14,2.79)	1.97 (0.96,2.98)	3.55 (1.15,6.01)	3.79 (2.09,5.53)	1.99 (1.23,2.77)
Lag5	2.19 (1.16,3.24)	4.08 (1.74,6.48)	3.43 (1.77,5.12)	2.09 (1.26,2.92)	1.95 (0.95,2.97)	3.99 (1.59,6.45)	3.57 (1.87,5.31)	1.97 (1.21,2.75)
Lag0–1	1.97 (0.83,3.11)	4.50 (1.94,7.23)	4.59 (2.74,6.48)	2.07 (1.16,2.99)	1.41 (0.31,2.52)	4.59 (1.87,7.38)	4.95 (3.01,6.92)	1.87 (1.02,2.71)
Lag0–2	2.21 (1.04,3.39)	4.41 (1.73,7.17)	5.01 (3.09,6.96)	2.25 (1.31,3.19)	1.95 (0.81,3.11)	4.33 (1.53,7.22)	5.40 (3.39,7.45)	2.26 (1.39,3.14)
Lag2–5	2.54 (1.36,3.73)	4.35 (1.64,7.13)	5.04 (3.12,7.01)	2.49 (1.55,3.43)	**2.23 (1.04,3.43)**	3.67 (0.76,6.65)	**5.70(3.59,7.85)**	**2.31 (1.42,3.22)**
Lag0–5	2.58 (1.36,3.81)	4.41 (1.62,7.28)	5.01 (3.02,7.04)	2.49 (1.51,3.47)	1.77 (0.53,3.02)	3.81 (0.75,6.97)	5.64 (3.43,7.90)	2.03 (1.09,2.98)

CVD, Cardiovascular disease; IHD, ischemic heart disease; HF, heart failure. Adjusted models are obtained by controlling wind speed, dew point, temperature, and holidays. The most effective lag for each disease is shown in Bold form. Data are shown as percent change (95% Confidence interval).

According to the results of two-pollutant models (Table 4), the number of IHD and cerebrovascular disease hospitalizations decreased after controlling for PM_10_ and SO_2_ and increased after controlling for NO_2_ and O_3_. For HF, a small decrease in percent changes was observed after controlling for SO_2_, NO_2_, and O_3_, whereas a slight increase in percent changes was observed after controlling for PM_10_. Additionally, the number of total CVD increased after controlling for SO_2_, NO_2_, and O_3_, whereas a slight declined was observed in the percent change after controlling for PM_10_. The results remained robust to the inclusion of the co-pollutants in models and were still significant.

**TABLE 4:** Adjusted percentage changes in co-pollutants and multiple pollutants per 1 mg/m^3^ increase in the concentration of CO for total and cause-specific CVDs.

	IHD	HF	Cerebrovascular disease	Total CVD
+O_3_	2.50 (1.22,3.80)	4.54 (2.07,7.07)	6.47 (4.16,8.84)	2.62 (1.65,3.60)
+NO_2_	2.53 (1.31,3.78)	4.47 (2.02,6.98)	6.08 (3.90,8.31)	2.40 (1.47,3.34)
+SO_2_	2.13 (0.89,3.39)	3.97 (1.46,6.54)	5.62 (3.41,7.86)	2.42 (1.49,3.37)
+PM_10_	2.05 (0.69,3.44)	4.66 (2.11,7.27)	4.29 (1.95,6.70)	1.98 (0.95,3.01)
Multiple pollutants	2.74 (1.38,4.12)	4.28 (1.78,6.78)	6.47 (4.04,8.96)	2.67 (1.64,3.71)

O_3_, ozone; NO_2_, nitrogen dioxide; SO_2_, sulfur dioxide; PM_10_, Particulate matter with an aerodynamic diameter <10 μm; CVD, Cardiovascular disease; IHD, ischemic heart disease; HF, heart failure. For the most effective lag of each disease, effects were controlled by time-varying meteorological (temperature, wind speed, and dew point) and holiday variables and additionally each of the pollutants in two pollutant models. In multiple pollutants models, latent factors obtained by exploratory factor analysis of NO_2_, O_3_, SO_2_, and PM_10_ added to models. Data are shown as percent change (95% Confidence interval).

Additionally, the results for multiple-pollutant models are shown in Table 4. The percent changes in daily hospital admissions per 1 mg/m^3^ increase in CO concentration in multiple-pollutant models for IHD, HF, cerebrovascular disease, and total CVDs were 2.74(1.38,4.12), 4.28(1.78,6.78), 6.47(4.04,8.96), and 2.67 (1.64,3.71), respectively, which shows a growth in the percent change in IHD, cerebrovascular disease, and total CVD and reduction in HF.

**FIGURE 1:**
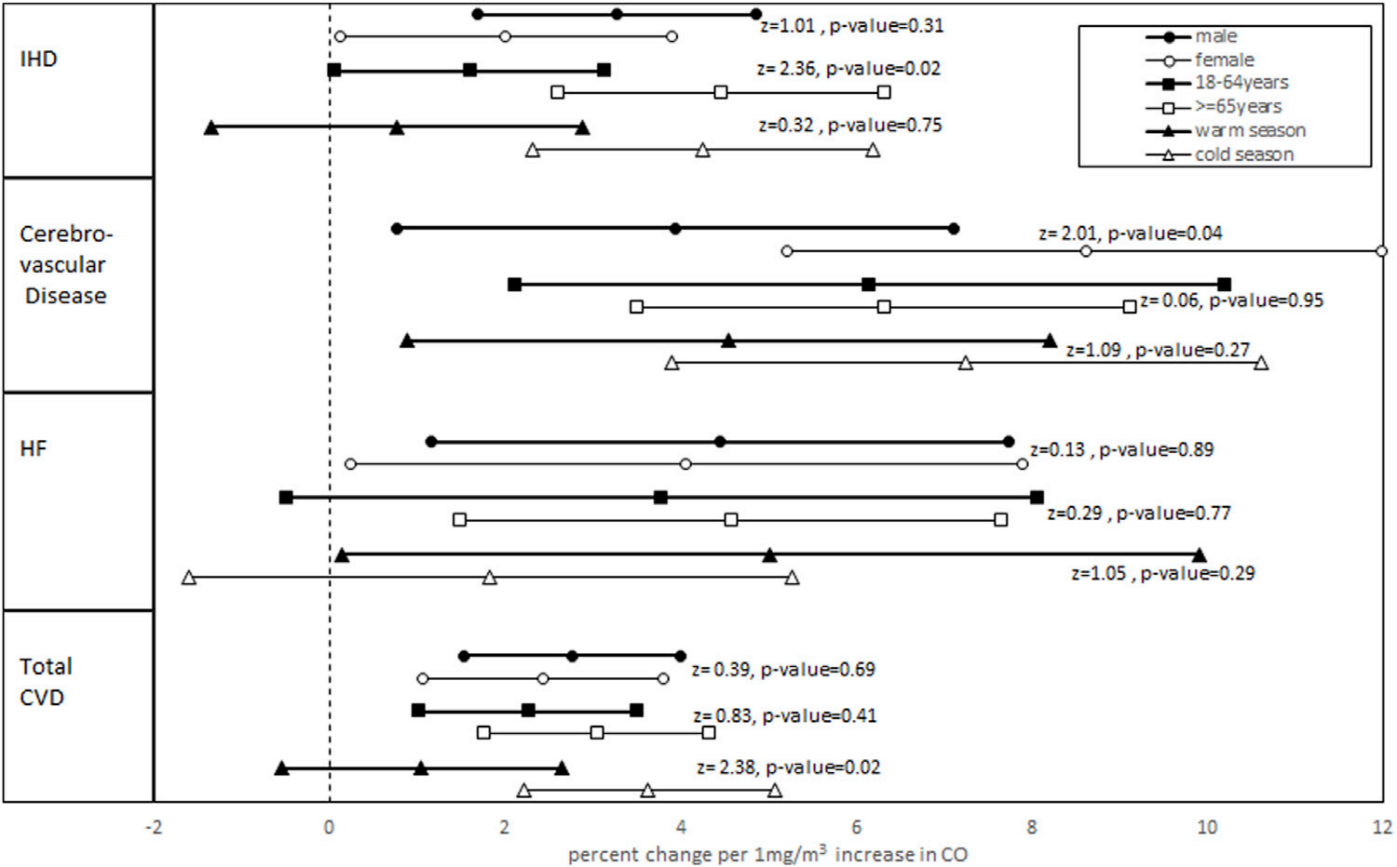
Adjusted percent change and 95% confidence interval in daily hospital admissions for total and cause-specific CVDs per 1 mg/m^3^ CO increase stratified by age groups, sex, and seasons. (Z-statistics and relevant p-values for comparing subgroups are shown).

Figure 1 summarizes the results derived from the stratification analyses. The associations between CO concentrations (lag0) with HF and CO concentrations (lag 2–5) with IHD, cerebrovascular disease, and total CVDs varied by age, sex, and season. For IHD, except the warm season, the results remained significant and became stronger for male patients or patients aged >= 65 years and for the cold season. Also, in IHD patients, a statistically significant difference was observed between age groups. For HF, except for the cold season and younger age group, the results were still significant in other subgroups. For cerebrovascular disease, no significant change was observed in subgroup analysis; however, in female patients and in the cold season, the percent changes became stronger, whereas in other subgroups, the associations were decreased. Also, there was a significant difference between the percent change in both male and female patients. The results for the total CVDs remained significant within all subgroups except for the warm season. Additionally, for male patients, the elderly age group, and cold seasons, there was an increase in the percent change, and in other subgroups, a decrease in the percent change was observed. Also, the difference between two seasons was statistically significant.

**FIGURE 2:**
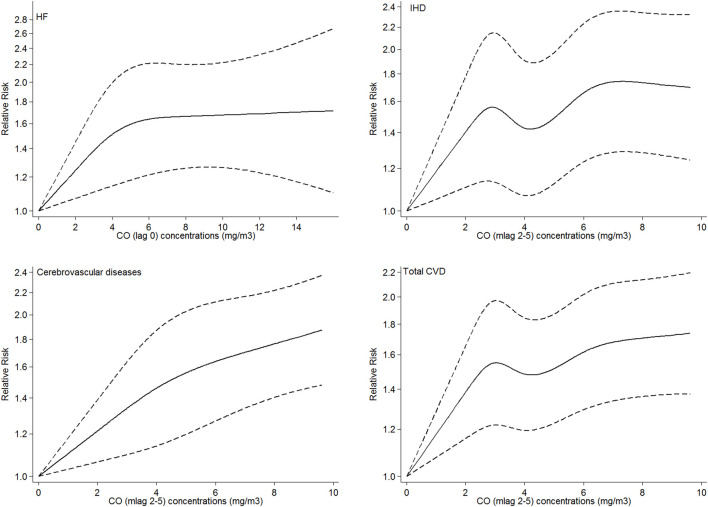
Adjusted exposure–response curves of exposure to CO with hospital admissions for HF, IHD, cerebrovascular disease, and total CVDs. The black solid lines represent the relative risk of hospital admissions, and the dotted lines represent the corresponding 95% confidence intervals. For each disease the most effective lag was considered.

Figure 2 shows the adjusted exposure–response relationship curve of the CO concentrations with total and cause-specific CVD admissions. For HF and cerebrovascular disease, three knots were selected according to AIC, while for IHD and the total CVD, five knots were considered. The estimated exposure–response relationships of the CO concentrations with HF admissions showed a linear relationship with a sharp increase in the dose–response function at lower concentrations (0–5 mg/m^3^) and a smooth increase at higher concentrations. For cerebrovascular admissions, a sharp linear trend was observed, while the exposure–response curve of the CO concentrations with IHD and total CVD admissions demonstrated a non-linear relationship with a sharp increase in the dose–response function at lower concentrations (0–2.5 mg/m^3^). Then, a slight decrease in the relative risk was observed within the concentrations (2.5–4 mg/m^3^), followed by a moderate increase between concentrations (4–6 mg/m^3^) and a slight increase at higher concentrations. The non-linearity of relationships was not significant for both HF and cerebrovascular disease according to the LRT while accepted for both IHD and total CVD.

## 4 Discussion

In the current study, in a time-series framework, we investigated the association between the ambient CO and hospitalizations for CVDs, using Poisson’s regression or negative binomial regression models and applying the data extracted from the CAPACITY study for patients aged 18 years or more. The mean concentration of CO in our study was 4.5 ± 2.3 mg/m^3^, which was a little higher than the recommended standard of 4 mg/m^3^ (Evangelopoulos et al., 2020) and higher than that in other studies such as Vahedian et al. (2017) and Xu et al. (2021).

Furthermore, we found that short-term exposure to ambient CO significantly increased the percent changes in hospital admissions related to cause-specific and total CVDs. Increasingly, scientific evidence has indicated that outdoor CO exposure is related to CVD hospital admissions and mortality. For example, Lee et al. (2020) in an updated systematic review and meta-analysis evaluated the short-term effects of CO on IHD hospital admissions or mortality. Their combined estimate across 26 studies demonstrated the risk ratio of 1.05 [with 95% CI of (1.02, 1.09)] for IHD per 1 mg/m^3^ increase in CO. In another study in Shiraz, Iran, in a long-term observational period, a significant association between CO and CVD-related hospital admissions was observed [RR = 1.52 (1.16, 1.99)] (Soleimani et al., 2009). Bell et al. (2009) found a positive and statistically significant association between the same-day CO concentration and increased risk of hospitalization for multiple CVD outcomes in US urban counties. Additionally, CO levels were associated with cerebrovascular diseases to a greater extent compared to any other CVDs, which was not seen in other studies. In contrast to our study, the results of Phosri et al. (2019) were not significant for cerebrovascular disease.

The largest estimated effects on IHD, cerebrovascular disease, and total CVDs in our study were observed in the moving average of lag2–5. Our results are almost consistent with a recent study conducted on older adults in Guangzhou, China, during 2016–2019, showing that each IQR increase in lag0–4 days of exposure to CO was significantly associated with a 4.53% increase in odds ratio of hospital admissions for sequelae of stroke (Wang et al., 2022). Motessadi Zarandi et al. (2022), in a time-series analysis carried out in Tehran, Iran, demonstrated that total CVD and IHD admissions were associated with CO for each 1 mg/m³ increase at lags of 6 and 7 days, which is close to our study. Unlike our study, a multicity time-series study conducted by Liu et al. (2018) in 272 major cities in China from January 2013 to December 2015 demonstrated that with a 1 mg/m³ increase in average CO concentrations at lag0–1, significant increases in mortality by 1.12% (0.42–1.83) for CVDs, 1.75% (0.85–2.66) for IHD, and 0.88% (0.07–1.69) for stroke will occur. Also, a time-series study in Bangkok, Thailand, from January 2006 to December 2014 demonstrated a 6.69% (4.33, 9.11) increase in CVD admission at lag0–1 day per 1 mg/m^3^ increase in CO (Phosri et al., 2019). Furthermore, in our study, for HF, the RR peaked at lag0, which is in agreement with a case–cross-over study conducted in Pennsylvania, United States, during 1987–1999 and showed that an IQR increase in the CO level at the hospitalization day led to a 4.55 (3.33, 5.79) percent increase (and 95% confidence interval) in the rate of hospital admissions for HF (Wellenius et al., 2005).

The subgroup analysis showed that although percent changes varied in different subgroups, only the results of the warm season in IHD and total CVDs and the cold season and younger age group in HF admissions were not more statistically significant. Additionally, our findings show significant differences between age groups in IHDs, between sex for cerebrovascular disease, and between seasons for total CVDs. A time-stratified case–cross-over study in China showed that a 1 mg/m^3^ increase in a 2-day moving average of CO concentration led to a rise of 2.8% (2.2, 3.3%) and 3.0% (2.4, 3.6%) in daily hospital admissions for CVD and IHD, respectively, which did not significantly differ within sex and age groups and was in contrast to our study (Li et al., 2018). Unlike our study, the associations did not vary substantially for city, region, and demographic characteristics such as age and sex in Liu et al. (2018). The findings of Xu et al. (2021), Motessadi Zarandi et al. (2022), and Wang et al. (2022) were not in agreement with our study and did not vary for sex and age groups. In contrast to our study, Xu et al. (2021) showed that CO exhibited a stronger association with mortality for IHD during the warm season. Whereas the results of stratification analysis in Phosri et al. (2019) and Vahedian et al. (2017) were consistent with the present study, and the elderly age group seemed to be the most affected group by CO.

Moreover, the results were controlled for other pollutants in the two- and multiple-pollutant models and remained robust. Although Liu et al. (2018), Li et al. (2018), Bell et al. (2009), and Motessadi Zarandi et al. (2022) adjusted other pollutants by only two pollutant models and did not use multiple-pollutant models, the same as our findings, their results were robust to controlling for other pollutants. Unlike our study, the result of Xu et al. (2021) did not remain robust when adjusted for SO_2_.

Additionally, in our study, the adjusted exposure–response curves demonstrated that the association between the CO concentration and IHD and total CVDs was partly linear, and the relative risk decreased in some regions, yet there is no obvious threshold below which CO exposure has no effects on CVDs. Even for decreasing regions of the curves, CO showed noticeable changes in relative risks. The same results were observed in Li et al. (2018), while the results of Xu et al. (2021) and Wang et al. (2022) were somehow different. In Xu et al. (2021), descending-shaped curves tended to become non-linear at higher CO concentrations, and in Wang et al. (2022), there was no evidence of non-linearity.

An increase in CO levels causes the oxygen-binding sites in hemoglobin to become occupied by CO and a decrease in the oxygen-carrying capacity (Yang et al., 1998). Little do we know about the exact pathophysiological mechanisms of acute adverse effects of CO exposure on cardiac patients; however, results of different studies account it for CVD patients. Also, we are not able to identify the mechanism for sex and age group disparity *via* a time-series study. Therefore, toxicological and population studies on exposure to mixtures of air pollution may determine the biological mechanisms that caused adverse health effects due to CO exposure.

### 4.1 Strengths and limitations

Our study had various strengths. First, our study was a part of the CAPACITY study, which is one of the few large-scale studies in this region designed to investigate the health-related effects of air pollution. Second, the study approximately covered most of the CVD admissions in Isfahan. Third, we investigated the potential association between ambient CO and total and cause-specific CVD admissions for different lag periods, age groups, seasons, and sexes. In addition, we considered the effect of other pollutants and meteorological data, and we investigated the adjusted exposure–response relationships.

However, our study had to deal with some limitations. First, as a time-series study, ecological bias is inherent. We had to consider the average air pollution over different stations since various parts of the city were exposed to different amounts of air pollution and the time people spent in each area varied greatly. Additionally, non-adequate pollution registration stations and unmeasurable distances from the stations may lead to measurement errors and exposure rate errors. Therefore, we were not able to quantify the exposure of individuals precisely. Second, in areas where CO is released alongside many other pollutants, it is almost impossible to undervalue the similar effects of other pollutants on CVDs. Although we adjusted the effects of other pollutants in our models, it is difficult to establish an isolated association. Third, although the GBD study denoted that indoor air pollution is one of the highest risk factors for all deaths worldwide, we did not consider CO exposure from indoor sources. Outdoor pollution may be approximately similar for residents who occupy the same area. Reversely, indoor pollution can be varied depending on the lifestyle of the people and the different air-conditioning systems in each house. In our study, no data regarding indoor air monitoring were accessible, and further studies may consider the effect of indoor air pollution on CVD-related hospital admissions. Ultimately, we were not capable of controlling potential individual confounders such as socioeconomic status, occupation, eating patterns, smoking, and other risk factors corresponding to CVDs that may impact hospital admissions.

## 5 Conclusion

Our findings have annotated that short-term exposure to ambient CO significantly increased the risk of hospitalizations for total and cause-specific CVDs in Isfahan, Iran. Furthermore, noticeable effects were observed for IHD in the elderly age group, for cerebrovascular disease in the female patients, and for total CVDs in the cold season. Additionally, multiple-pollutant adjustments raised the effects except for HF. Also, non-linear effects were observed for IHD and total CVDs. A long-period study considering confounders like socioeconomic status and lifestyle status such as eating habits, smoking, and physical activity that may affect the cardiovascular hospital admissions may better demonstrate the clear effect of CO and other air pollutants on CVDs.

## Data Availability

The raw data supporting the conclusions of this article will be made available by author Nizal Sarrafzadegan (nsarrafzadegan@gmail.com), without undue reservation.
